# Mass screening for chronic kidney disease in rural and remote Canadian first nations people: methodology and demographic characteristics

**DOI:** 10.1186/s40697-015-0046-9

**Published:** 2015-03-19

**Authors:** Barry Lavallee, Caroline Chartrand, Lorraine McLeod, Claudio Rigatto, Navdeep Tangri, Allison Dart, Audrey Gordon, Stephanee Ophey, Paul Komenda

**Affiliations:** Diabetes Integration Project, Winnipeg, Canada; University of Manitoba, Centre for Aboriginal Health Education, Winnipeg, Canada; Manitoba Renal Program, Winnipeg, Canada; Department of Medicine, Section of Nephrology, University of Manitoba, Winnipeg, Canada; Seven Oaks General Hospital Research Centre, Winnipeg, Canada; Children’s Hospital Research Institute of Manitoba, Winnipeg, Canada; Seven Oaks General Hospital Kidney Health Program, 2300 Mcphillips Street, 2PD12, Winnipeg, MB R2V 3M3 Canada

**Keywords:** Chronic kidney disease, Screening, First nations health, Risk prediction

## Abstract

**Background:**

Screening the general population for Chronic Kidney Disease is not currently recommended.. Rural and remote Canadian First Nations people suffer a disproportionate burden of Kidney Failure. The ***Fi***rst ***N***at***i***ons Community Based ***S***creening to Improve Kidney ***He***alth and Prevent ***D***ialysis (***FINISHED***) project intends to test the hypothesis that a mobile, mass screening initiative available to all First Nations people 10 years of age and older residing in rural and/or remote communities, is feasible, will improve health outcomes and is cost effective.

**Objectives:**

The objective of this manuscript is to describe the key elements required to design, implement and evaluate such a program and describe key characteristics of our screened cohort.

**Design:**

Methods and cohort description.

**Setting:**

11 First Nations communities within 2 Tribal Councils in Manitoba, Canada.

**Patients:**

All First Nations individuals between the ages of 10–80 living in the 11communities were eligible for the screening initiative.

**Measurements:**

Screening Rates achieved within communities.

**Methods:**

An interdisciplinary team partnership was established between the Diabetes Integration Project and the Manitoba Renal Program. Stakeholder consultation was obtained and protocols developed to mass screen community members using point of care testing equipment. All people screened were risk stratified, counselled and referred to nephrologists as required in real time, based on risk.

**Results:**

As of August 31, 2014, 1480 people in 11 communities over 2 Tribal Councils have been successfully screened. A mean screening rate of 21% of all community members eligible (aged 10–80) has been achieved. All patients at intermediate or high risk of kidney failure have been seen by nephrologists within 1 month of screening.

**Limitations:**

Long term outcomes of kidney failure rates not assessed for at least 5 years. Alternative public health initiatives to reduce kidney failure not investigated.

**Conclusions:**

Point of care mass screening, real time risk prediction and counselling of First Nations people at high risk of Kidney Failure is feasible in rural and remote communities. Further analysis of this cohort will describe theepidemiology of CKD in these communities, and test the cost effectiveness of this strategy.

**Electronic supplementary material:**

The online version of this article (doi:10.1186/s40697-015-0046-9) contains supplementary material, which is available to authorized users.

## Abrégé

**Contexte:**

Présentement, le dépistage universel systématique des maladies rénales chroniques est une pratique ni recommandée ni souhaitée. Les membres des Premières Nations du Canada qui vivent en région rurale ou éloignée sont aux prises avec un fardeau d’insuffisance rénale beaucoup plus lourd que le reste de la population. Le projet de dépistage des maladies rénales et de prévention de la dialyse des communautés des Premières Nations, l’initiative FINISHED (pour *First Nations Community Based Screening to Improve Kidney Health and Prevent Dialysis*), a pour but de tester l’hypothèse suivante : le dépistage universel des membres des Premières Nations de 10 ans ou plus et vivant en régions éloignées ou rurales au moyen d’une unité de service mobile est un projet faisable, rentable, et qui aura des répercussions positives sur la santé des communautés desservies.

**Objectifs de l’étude:**

L’objectif principal de cet article est, d’une part, la description détaillée des principaux éléments nécessaires à la conception, l’implémentation et l’évaluation d’un programme de cette envergure, et d’autre part, la production de données préliminaires décrivant les caractéristiques de base de la cohorte dépistée.

**Type d’étude:**

Éléments méthodologiques et description de la cohorte.

**Lieu de l’étude:**

11 communautés des Premières Nations provenant de 2 conseils de bande du Manitoba, au Canada.

**Patients:**

Tous les membres des communautés des Premières Nations sélectionnées, âgés de 10 à 80 ans, étaient admissibles à l’initiative de dépistage.

**Mesures:**

Taux de dépistage atteint au sein des communautés.

**Méthodes:**

Une équipe multidisciplinaire, issue d’une collaboration entre le *Diabetes Integration Project* et le *Manitoba Renal Program* a été formée pour le projet. Après une vaste consultation des parties prenantes, les protocoles et les lignes directrices de fonctionnement du dépistage universel des membres de la communauté, utilisant l’équipement de dépistage disponible aux points de service, ont été développés. Les personnes dépistées ont été classées selon leur niveau de risque; après consultation, un suivi avec un néphrologue a été initié, si nécessaire au moment de l’évaluation.

**Résultat de l’étude:**

Au 31 août 2014, 1480 personnes provenant des 11 communautés des 2 conseils de bande ont été dépistées avec succès. Le taux de dépistage moyen de l’ensemble des membres admissibles (âgés de 10 à 80 ans) atteint est de 21%. Tous les patients aux prises avec un risque élevé ou modéré d’insuffisance rénale ont été rencontrés par des équipes multidisciplinaires en néphrologie, à l’intérieur d’une période d’un mois suivant le dépistage.

**Limites de l’Étude:**

Les répercussions à long terme des taux d’insuffisance rénale n’avaient pas été évaluées depuis un minimum de 5 ans. Les initiatives de santé publique complémentaires visant à diminuer les taux d’insuffisance rénale n’ont pas été examinées.

**Conclusions:**

Il est faisable d’effectuer le dépistage universel, la prévision du risque de maladie et la consultation en temps réel des membres des Premières Nations à haut risque d’insuffisance rénale, à partir de points de service, en région rurale ou éloignée. Une analyse plus poussée de cette cohorte pourra faire ressortir les données épidémiologiques actuelles sur la maladie rénale chronique au sein des communautés visées, et permettra d’évaluer la rentabilité de cette stratégie, dont le but est la réduction du fardeau d’insuffisance rénale, et des répercussions engendrées par les complications qui en découlent.

## What was known before

**First Nations people living in many rural and remote communities suffer from a disproportionate risk of Chronic Kidney Disease and Kidney Failure associated with Type 2 Diabetes. Some of this is likely is likely attributable to poor access to routine primary and specialty preventative care.**

## What this adds

**A mobile point of care mass screening and risk based counselling program for Chronic Kidney Disease is feasible in rural and remote First Nations communities. Meaningful partnerships between First Nations communities, government, experts in indigenous health and nephrology specialist teams ensure the success of delivering these types of innovative initiatives.**

## Background

Patients with Chronic Kidney Disease (CKD) and its end stage of Kidney Failure (KF) have poor health outcomes and utilize a disproportionate amount of health care resources [1-3]. Patients with CKD are at high risk of early death and disability and rural KF patients may often require relocation to receive facility based hemodialysis [4-6]. Despite the high costs of providing this therapy, health outcomes and quality of life are frequently very poor, with over half of KF patients dying in the first five years of treatment. Routine, mass screening of the general population is not desirable or recommended, but targeted screening and treatment of patients at risk with hypertension or diabetes has been found to be cost effective in a variety of settings [7,8].

The province of Manitoba has amongst the highest incidence and prevalence of KF in Canada, driven by a disproportionate burden of diabetes and CKD in First Nations communities [9,10]. Preventative health care delivery and screening for modifiable disease presents a unique challenge in that many First Nations people often reside in remote communities. Despite persistent health disparities and the challenges of living in poverty, First Nation communities continue to receive poor primary and specialty care services [11,12]. Other features such as a younger population (50% < 19 years of age) and inequities in the social determinants of health present additional challenges [13]. Compounding these risks, First Nations individuals have reduced rates of kidney transplantation and living kidney donation, in part due to a strong family history of diabetes and overall reduced access to health care [14].

First Nations children have a 2-6-fold higher risk of CKD than non-First Nations children in Canada [15,16]. Manitoba also has the highest rates of youth onset type 2 diabetes in Canada (12 fold higher than any other province) [17]. This is especially concerning, as youth onset diabetes has been shown to be associated with rapid progression of CKD leading to KF in early adulthood [18]. First Nation children also often have an earlier onset of risk factors for CKD including high rates of obesity, hypertension, and metabolic syndrome [18]. They often live in poverty and are faced with food insecurity, inter-generational trauma, poor housing, have high levels of stress and emotional distress [19,20] and have low educational attainment [21,22].

It is clear that a new paradigm is urgently needed to address the growing number of First Nations people afflicted with complications of Type 2 Diabetes such as CKD and KF. The ***Fi***rst ***N***at***i***ons Community Based ***S***creening to Improve Kidney ***He***alth and Prevent ***D***ialysis (***FINISHED***) project intends to test the hypothesis that a mobile, mass screening initiative available to all First Nations people 10 years of age and older residing in rural and/or remote communities, is feasible. The research questions of this project will include reports on the epidemiology of CKD in adult and pediatric populations screened, the modelled cost effectiveness of this initiative and the long term outcomes of individuals screened. The primary objective of this manuscript is to describe in detail the key elements in the design, implementation of such a program and provide preliminary data on the baseline characteristics of our screened cohort.

## Methods

### Stakeholder engagement, governance structure and multidisciplinary team components

To lead this project, a multidisciplinary project team was assembled consisting of indigenous nurses and a physician with extensive experience in community based screening in First Nations communities (Diabetes Integration Project) [23], adult and pediatric clinician scientists with expertise in nephrology and epidemiology, a project manager, and a communications specialist. Indigenous protocols for public health interventions and research were made a priority within this project team and all data collected maintained strict adherence to OCAP principles (Ownership, Control, Access, and Possession) [24].

We conducted extensive stakeholder engagement in the design, conduct and evaluation component of the FINISHED screening project. This engagement included iterative focus group meetings with individual small gatherings as the project plan was developed and refined in continuous feedback loops with Health Canada and Provincial Renal Program decision makers, Tribal Council Leaders, and Health Director and Chief and Council members of individual communities. Adhering to community protocols, Elders have participated in the design and formative reviews through the life of this initiative. Their participation has been instrumental in opening dialogue around the importance of traditional medicines and ceremony in the context of health and healing for community members.

**Figure 1:**
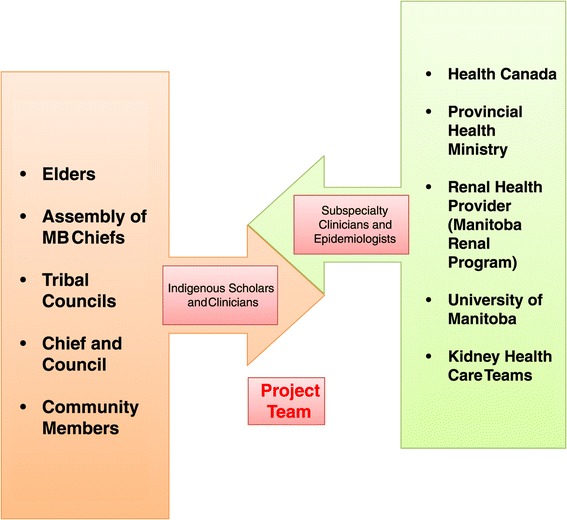
**Relevant stakeholders.**

Canadian First Nations people represent a unique population with regards to history, culture, language, values beliefs and relationships. At the core, critical components to lead a screening campaign such as this must include interprofessional partnerships beginning with indigenous clinicians and scholars working alongside epidemiologists, subspecialty kidney health care teams, and engaged levels of government (Figure 1). While we obtained Research Ethics Board approval from the University of Manitoba for this screening program, this alone is insufficient to conduct a public health initiative of this scope and magnitude in First Nations Communities.

Government participation is complex, as on-reserve care of First Nations people is the responsibility of Health Canada, under the First Nations Inuit Health portfolio. However, the provincially funded health system most often bears the responsibility to provide subspecialty services such as CKD and diabetes prevention and resource intense services such as dialysis. Funding for this initiative was provided by Health Canada, and First Nations governments played a key role in the development, delivery and implementation of FINISHED including the Assembly of Manitoba Chiefs, Tribal Councils, and individual community chief and councils.

This project was led by two key partner organizations: the Diabetes Integration Project and the Manitoba Renal Program. The Diabetes Integration Project partners brought expertise in the use of point of care technology and delivery of care in First Nations Communities. The Manitoba Renal Program nephrologist team members provided expertise in appropriate screening and risk prediction methods and ensured that all intermediate and high risk patients were seen in multidisciplinary CKD prevention clinics in a timely fashion. A steering committee consisting of key stakeholders from the Provincial and Federal Governments, Tribal Councils and Provincial Renal Program met quarterly to provide additional insight and suggestions to help guide the project governance.

### Screening setting

As this type of large scale screening and real time risk prediction and counselling had not been undertaken before, a representative sample of different types of communities to screen was preferred. We sought a mix of communities with different distances to urban settings, with some having road access and some accessible primarily by air. Communities of heterogeneous size in terms of geographic area and population were represented. Each of these factors presented unique challenges for operationalizing screening on a large scale.

### The screening process

An effective and efficient mass screening program in rural and remote First Nations Communities must be capable of high throughput, and yet still accurately depict individual risk, readily translatable from non-physician clinicians at the point of care, in the field to patients. The process must be designed in such a way that use of equipment, technology and human resources are deployed optimally and appropriate to the varying strengths and challenges found at the community level. Initially, screening teams consisted of a registered nurse, a licensed practical nurse and a health care aid with the relevant clinical qualifications and knowledge of indigenous customs, culture and language. As experience was gained and protocols refined, health care aids were trained to conduct point of care testing and obtain consents and teams were pared down to consist of a registered nurse and health care aid only through a “delegation of function”.

A process engineer was consulted to aid in the design of an efficient screening process. Informed consent was obtained from each patient after watching a short video on the purpose and process for kidney health screening and having the opportunity to ask questions to one of the screening nurses. Consent was obtained for the screening itself, in addition to the ability for the project team to investigate administrative sources of lab and health care utilization data into the future to better monitor patients with CKD. This will allow tracking of patient health outcomes and use of the health care system through longitudinal studies. Consent for children <18 was obtained from their legal guardian prior to screening and assent was obtained from the children themselves.

Screening took place at a variety of different locations including Health Centres, Nursing Stations and schools, depending on the community. In the field, baseline demographic data collected included personal health number, date of birth, and name of home community. A mean of six blood pressures were taken (BPTru Medical Device, Coquitlam, BC) according to best practice as outlined by the Canadian Hypertension Education Program (CHEP) [25]. Height, weight and body mass index were recorded on each patient. Finger prick droplet blood samples were taken (0.1 cc) and analyzed for blood chemistry including creatinine, estimated Glomerular Filtration Rate (eGFR) derived from the Modification of Diet in Renal Disease 4-variable equation [26], calcium, phosphate, albumin and total carbon dioxide (Picollo Xpress©, Abaxis, Union City, CA), and a separate droplet was collected for glycosylated hemoglobin and urine sample was analyzed for urine albumin to creatinine ratio (DCA Vantage ©, Siemens, Erlangen, Germany). All point of care testing equipment was subjected to a comprehensive quality assurance process to ensure the results obtained achieved precision and accuracy calibrated to a main frame laboratory. The internal quality assurance process was completed on a daily basis utilizing human serum samples with pre-defined performance limits and monitored by an external reference method laboratory (Canadian External Quality Assurance Laboratory; Vancouver, Canada).

**Figure 2:**
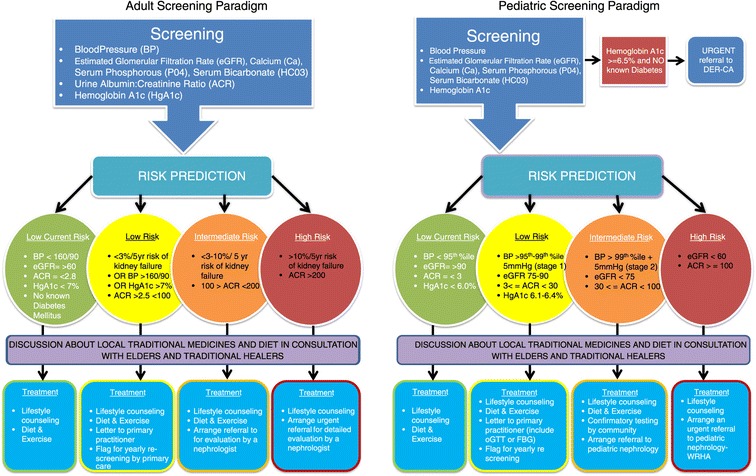
**Adult screening paradigm.**

All patient consents, demographic, blood pressure and biochemical data were entered at the point of care on to a tablet computer (iPad3, Apple, Cupertino, CA), using a secure customized application created specifically for this project by an in-house computer programmer. The data entered would automatically calculate a patient’s five-year risk of kidney failure as calculated by the Kidney Failure Risk Equation (KFRE) [27] for adult patients screened. Using data from the KFRE and a few other parameters such as level of proteinuria, adult (>18 yo) patients were assigned to a category of risk of low, intermediate or high corresponding to their 5 year risk of kidney failure and other parameters such as level of proteinuria for those with eGFR >60 ml/min/m [2]. (Figure 2). Risk based counselling scripts were developed and delivered by the screening teams in real time (Additional file 1).

**Figure 3:**
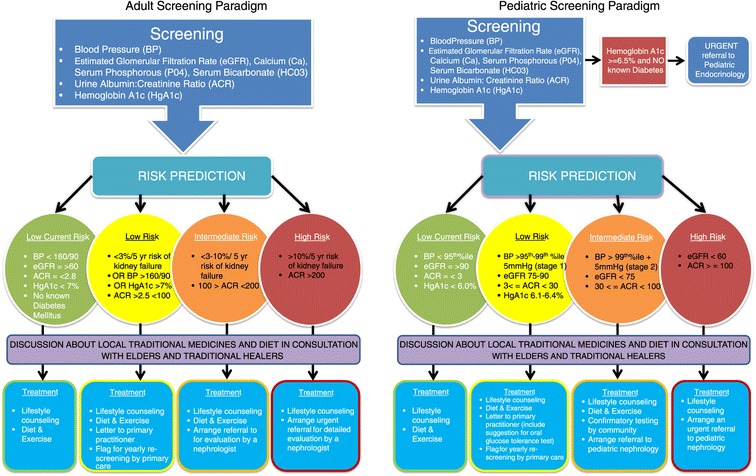
**Pediatric screening paradigm.**

As the KFRE has not been validated in children, pediatric (<18 yo) patients followed a separate algorithm designed in collaboration with pediatric nephrologist and endocrinologist partners based on best practice and review of current guidelines and literature. These referral pathways were based on clinical parameters including eGFR (calculated by the Schwartz formula [28] proteinuria), and blood pressure percentiles [29] (Figure 3). Children with a HgbA1C > 6.5% were also referred to the Diabetes Education Resource for Children and Adolescents (DER-CA) in Winnipeg.

Based on risk category generated from our algorithms, patients and guardians would receive a risk-based counselling session in real time by the screening nurses. A pre-approved script developed by the multidisciplinary project team for pediatrics was developed for this purpose (Additional file 2). Primary Care providers were sent the results of screening and plan for each patient including specific treatment recommendations for children, although all intermediate and high-risk patients were also immediately referred to a nephrologist directly from the screening results. All nephrology referrals derived from the project were seen within one month of screening. Occasionally, limitations of the point of care equipment in measuring urinary albumin excretion would not allow the team to assign an exact estimate of risk in real time (<5% of cases). In these cases, a provisional risk was assigned to a patient and a urine sample was taken to send to one of the central laboratories for the sample to be re-run and patients were contacted the next week for their final risk assessment. Finally, all adult patients were consented to answer an additional paper based questionnaire exploring in further detail their medical history, social determinants of health and questions concerning their experience with personally mediated racism.

### Enhanced screening

Time permitting, a subset of adult patients (1:10) were randomly selected to undergo an “enhanced screening” arm of this intervention. This subset received phlebotomy and urine specimens were collected. Blood specimens were centrifuged and serum supernatant was aliquoted on site in the field, and cold chain was maintained until reaching the −80 freezers at the biobanking facility in Winnipeg on a weekly basis.

### Engagement protocols and feedback

Our team relied on and learned from the Diabetes Integration Project’s experience and expertise in gaining access to First Nation communities, specifically in the area of indigenous protocols and contacts for engagement. The principle of community participation was a primary goal of FINISHED. Engagement with Chief and Council, Tribal Council Leaderships, Assembly of Manitoba Chiefs, Federal and Provincial governments and Regional Health Authorities along with inclusion of elders, intense community consultations and dialogue were necessary to deliver a message that complications from CKD were often preventable. It was important that our health care team members (including nephrologists) try to participate in as many of the community engagement events as possible to deliver a message of hope and commitment to the project. Community participants, many for the first time, asked questions directly of physicians about general health concerns and those related to chronic kidney disease. Flexibility to change schedules, address community concerns and be open to the ongoing challenges communities face in their effort to improve health outcomes for members ensured a community centred approach.

A detailed set of Standard Operating Procedures was developed by the Project Leadership Team ensuring that proper protocols were followed within each community screened well in advance of proposed screening dates**.** Members of the Project Leadership Team (physicians, nurses, project manager) travelled to each community to present to the Chief and Council, Health Portfolio Members, Health Directors and the Aboriginal Diabetes Initiative Worker regarding the project’s purpose and processes, ask specific permission to screen members of the community and provide an interactive opportunity to provide feedback and suggestions. Following this, an advertised Community Town Hall meeting was arranged to present to the Community Members at large and provide opportunity for questions and feedback. To ensure the success of pediatric screening, presentations to the school administration, teachers, and eligible students were also made in several communities by the team, including a pediatric nephrologist. Within a month following these meetings, the screening teams would arrive in the community to begin their activities. Around this time, door hangers were hand delivered to each home reminding community members about location and timing of screening and a provincial and local radio stations were employed to encourage people to come to get screened.

Immediately following screening activities in the Community, reports were prepared for feedback to the Community Leadership. Reports included data on the number of patients screened, their demographics, screening results stratified by risk category and number of referrals made to nephrologists. These reports were provided to Community Leaders in paper forms and the members of the Project Leadership Team travelled back to each Community to provide a presentation and give the opportunity for Community stakeholders to ask questions regarding their results. Permission was obtained from each Community Leadership group to report on aggregate, anonymous data on behalf of the project.

## Results

All communities approached in the 2 Tribal Councils screened agreed to participate in the program. We have successfully screened 11 communities representing 1480 patients using the methodology described in this manuscript. The communities represented two Tribal Councils within Manitoba and were selected to represent a mix of large and small communities ranging in size from 56 – 2876 people greater than the age of 10 (Table 1). Four of these communities were very remote an only accessible by air for the majority of the year. The remaining seven communities were accessible by road.

**Table 1:** Baseline characteristics of screened cohort (n = 1480 as of August 31, 2014**)

**Community**	**Age (mean, SD)**	**Sex (% female)**	**Fly-in or road access**	Population of community eligible to screen (>10 yo)*	**Community members screened (N)**	**# of days of screening**
1	45.5 (16.9)	54.9%	Road	421	61	14
2	44.8 (17.9)	59.3%	Road	56	25	4
3	39.7 (15.4)	54.4%	Road	365	170	20
4	39.4 (16.9)	57.1%	Road	554	141	14
5	40.0 (16.8)	64.6%	Fly-in	2876	302	58
6	35.8 (20.6)	55.6%	Fly-in	703	195	18
7	36.4 (19.0)	49.5%	Road	379	94	9
8	35.5 (19.6)	68.5%	Road	1281	185	21
9	40.2 (20.4)	67.5%	Road	800	77	14
10	40.7 (17.5)	60.3%	Fly-in	2819	187	35
11	43.0 (14.7)	65.6%	Fly-in	1361	43	7

*Derived from Status Verification System (SVS) “On-Reserve” Population Totals Report, June 2014 projecting that 75% of population on Reserve is >10 years of age.

**Screening efforts to continue until December 2014.

We have thus far achieved screening rates ranging from 3.15 – 46.6% (of eligible members aged 10–80) as of August 31^st^, 2014. Screening efforts will continue until December 2014. The mean screening rate in fly-in remote communities was 12% (SD 9.44) while more accessible communities with road access achieved screening rates of 25.7% (SD 13.7). We have achieved 100% data capture as we have utilized point of care testing equipment and electronic data capture in real time. Table 1 describes baseline characteristics of each community screened in terms of age, accessibility, time spent screening and percent screened. To date, all patients deemed intermediate or high risk of kidney failure in both pediatric and adult populations received nephrology referrals and have been seen by a nephrologist in Winnipeg (our tertiary referral centre) within one month of screening. This initiative had no adverse effects on lengthening wait times for pediatric or adult nephrology clinics referred to as there was no wait list to see nephrologists prior to and during the screening initiative at referral centres. No major adverse events have been reported as a result of the screening initiative.

## Discussion

A mass screening initiative for Chronic Kidney Disease in rural and remote First Nations communities using state of the art point of care testing, electronic data capture, on site risk prediction and risk based counselling is feasible and acceptable to communities. This approach represents one potential option to improve chronic disease detection and treatment in at risk populations with poor access to primary care and social determinants of health compounding risks of poor outcomes associated with CKD.

We are aware of a number of CKD screening initiatives that have been conducted in a variety of jurisdictions worldwide. The National Kidney Foundation Kidney Early Evaluation Program (KEEP) has screened over 185,000 individuals in the United States targeting people over the age of 18 with hypertension, diabetes or a family history of CKD [30] yielding a wealth of novel epidemiologic data on early detection and surveillance of CKD [30,31]. These same screening efforts have been replicated in Japan, Mexico and elsewhere. The Korean National Health Screening performed a cross sectional mass screening study of the general population using eGFR and dipstick proteinuria on over 10 million participants in that country finding relatively low prevalence of CKD (6.15%) [32]. The Thai led screening and early evaluation of Kidney Disease (SEEK) study used stratified cluster sampling to mass screen 3459 subjects finding an overall CKD prevalence of 17.5%. In Canada, the Screening for Limb, I-Eye, Cardiovascular and Kidney Complications (SLICK) Program was implemented in 1999 to improve diabetes care for First Nations in Alberta. Nine hundred and eighty First Nation individuals living with diabetes in 44 communities underwent extensive monitoring and targeted treatment for diabetes complications demonstrating improvements in many clinical parameters including BMI, blood pressure, cholesterol and HbA1C [33,34].

In contemplating this initiative, our group performed a systematic review of the cost effectiveness studies for CKD screening [7]. These studies have used heterogeneous approaches to screening, mainly utilizing proteinuria or eGFR alone as a screening test. Not surprisingly, we found that screening those at risk of CKD as a result of documented hypertension or diabetes likely represented good value for money, whereas mass screening of the general population did not. Little cost effectiveness data exists for specific populations at risk such as First Nations people, or those with poor access to primary care services due to location of residence. We know that these populations under threat suffer from a disproportionate burden of kidney failure and other complications of type 2 diabetes and that this risk seems to be increasing over time [34,35]. The intent of this project is to report on the true epidemiology of CKD in rural, remote First Nations people and to ensure that those detected receive timely and seamless care within a health care system often not optimally suited to break down cross-jurisdictional barriers between federal and provincial providers.

There are several plausible explanations for the health disparities observed in Canadian First Nations communities. Structural racism continues to diminish and conceal the strengths found within the communities. The Indian Act and the socially constructed interventions like the residential school and sixties scoop contribute to the inter-generational oppression and challenges faced by First Nations who wish to attain equal health outcomes as settler Canada enjoys. Few Canadians realize these represent both historical and current racially constructed interventions aimed to diminish and demean First Nation identity; they continue to have devastating and brutal impacts on the health and healing of these communities [36].

While we have demonstrated that our approach is feasible, many limitations and questions remain. First, screening is very costly in more remote, fly-in communities driven primarily by travel costs, shipping of equipment and accommodations for screening teams. Turnout rates for screening can be low for a variety of unforeseen circumstances (e.g. structural challenges, deaths in community, competing events, local transportation logistics, mistrust of providers external to the communities and floods). Comprehensive multimedia communication with community members, flexible screening teams in terms of time and approach and engaging specific community members such as Aboriginal Diabetes Initiative workers are key to overcoming some of these challenges). However, in these cases, the cost per screened patient can increase tremendously. Work is currently underway to perform formal cost effectiveness analyses on the data derived from this project. Second, the project has been diligent in ensuring all intermediate and high risk referrals are seen in the tertiary care referral site (Winnipeg) within one month of screening. These consultations might be better accommodated with outreach clinics, or by telehealth that might improve patient satisfaction and comfort in future initiatives. It is possible that our risk prediction filters in adult and pediatric populations are imperfect, however the KFRE was derived and validated in Canadian populations including Aboriginals and race was not a factor in improving the equation’s performance. *The equation has subsequently been validated in 23 additional cohorts on five continents worldwide including over 700,000 individuals including Pima Indian populations* [37]*.* Pediatric algorithms were derived by pediatric nephrologists and endocrinologists according to review of the literature and best current practice. Third, while we have assumed that early detection of existing disease and appropriate referral of at risk patients to primary care or nephrologists commensurate with risk within communities represents the best value for money in attenuating downstream consequences of CKD. We have not, however, provided a comparator group here where an equal amount of resources were spent on building capacity within communities to employ a semi-structured, multi-faceted approach to combating diabetes and detecting complications. We are keenly aware of this limitation and are planning to test these two options using a cluster randomized controlled trial design examining short and long term surrogate outcomes for CKD.

## Conclusions

A community based mass screening initiative for CKD in rural and remote First Nations communities using point of care testing, electronic data capture, and real time risk prediction and patient counselling is feasible and acceptable to patients and communities. This screening initiative offers some insight into the prevalence of CKD and can be replicated to address different chronic diseases in rural, remote and urban communities. It remains unknown if this type of approach represents good value for money compared with other established community capacity building programs for diabetes prevention with demonstrated success in other jurisdictions. Further study on this question is required in the form cost effectiveness analyses which are underway, and community level cluster randomized controlled trials.

## Additional files

Additional file 1:
**Adults Scripts for Risk Based Counseling.**
Additional file 2:
**Pediatric Scripts for Risk Based Counseling.**
