# Supplementary material for: Mass screening for chronic kidney disease in rural and remote Canadian first nations people: methodology and demographic characteristics
Source: Can J Kidney Health Dis. 2015 Mar 19;2:9. doi: 10.1186/s40697-015-0046-9 (PMC4940863; doi:10.1186/s40697-015-0046-9)
Supplement: Additional file 1: — Adults Scripts for Risk Based Counseling. [file 40697_2015_46_MOESM1_ESM.pdf]

Adult Scripts for Risk Based Counseling

HIGH RISK

High Risk:

We are screening for Kidney Disease because
we know that if kidney disease/sick kidneys are found early, they can be treated with medications (pills) to keep them from getting sicker and to prevent the need for dialysis.

Sick kidneys can get sicker over time and they can get so sick that they no longer work. When very sick kidneys can't filter (or clean) blood like they should, then a person needs to go on dialysis. You may have heard the word dialysis before; dialysis is used to filter the blood and when a person uses dialysis they must go to the dialysis centre or hospital 3 times a week or get dialysis every night at home.

Your urine and blood tests today show that your kidneys might be sick. This means that you need to see a kidney doctor soon, so that they can check how sick they are and why they are sick. So the best thing we will do today is to make an appointment for you to be seen at a special Kidney Clinic in Winnipeg in the next 4-6 weeks.

At the clinic, a kidney specialist doctor will do more tests to see what is happening to your kidneys. After the tests, the kidney doctor will be able to explain to you what the problem is and will talk to you in detail about the best treatment choices to keep your kidneys from getting sicker. You will have a chance to ask lots of questions, and you will be followed closely by them from now on.

We will also send a letter to your main doctor or community nurse explaining the results of the screening tests today. You should follow-up with them as soon as possible to talk about eating good and being active these things are important for your health and for keeping your kidneys healthy.

(For WRTC area) Do you see a traditional healer? (If yes) you may want to talk with them about things you can do for kidney health or your overall health. If they give you medicine to use you may want to let the kidney doctor know that you are using traditional medicine.

Finally, I want to make sure that you understand that it is very important that you go to that kidney clinic appointment in Winnipeg. Not knowing about or not treating sick kidneys increases your chance of your kidneys getting very sick and having to use dialysis. We are going to work together to prevent your kidneys from getting sicker.

Adult Scripts for Risk Based Counseling

INTERMEDIATE RISK

Intermediate risk:

We are screening for Kidney Disease because we know that if kidney disease/sick kidneys are found early, they can be treated with medications (pills) to keep them from getting sicker and to prevent the need for dialysis.

Sick kidneys can get sicker over time and they can get so sick that they no longer work. When the kidneys can no longer filter (or clean) the blood like they should the person then needs to go on dialysis. You may have heard the word dialysis before, dialysis is used to filter (or clean) the blood, when a person needs dialysis they must go to the dialysis centre or hospital 3 times a week or get dialysis every night at home..

Your urine and blood tests today show that your kidneys might be sick. This means that you need to see a kidney doctor so that they can check how sick they are and why they are sick. So the best thing we will do today is to make an appointment for you to be seen at a special Kidney Clinic in Winnipeg in the next 3 – 6 months.

At the clinic, a kidney specialist will do more tests to see what is happening to your kidneys After the tests, the kidney specialist will be able to explain to you what the problem is, and will talk to you in detail about the best treatment choices to keep your kidneys from getting sicker and to help get your blood pressure under control

We will also send a letter to your main doctor or community nurse explaining the results of the screening tests today. You should follow-up with them as soon as possible to talk about eating good and being active, these things are important for your health and for keeping your kidneys healthy.

(For WRTC area) Do you see a traditional healer? (If yes) you may want to talk with them about things you can do for kidney health or your overall health. If they give you medicine to use you may want to let the kidney doctor know that you are using traditional medicine.

<u>Finally, it is very important that you go to that appointment in Winnipeg.</u>
Not knowing about or not treating sick kidney increases your chance of your kidneys getting very sick and you having to use dialysis. We want to work together with you to prevent your kidneys from getting sicker.

Adult Scripts for Risk Based Counseling

LOW RISK

Low Risk:

We are screening for Kidney Disease because we know that if kidney disease/sick kidneys are found early, they can be treated with medications (pills) to keep them from getting sicker and to prevent the need for dialysis.

Sick kidneys can get sicker over time and they can get so sick that they no longer work. When the kidneys can no longer filter (or clean) the blood like they should the person then needs to go on dialysis. You may have heard the word dialysis before, dialysis is used to filter (or clean) the blood, when a person needs dialysis they must go to the dialysis centre or hospital 3 times a week or get dialysis every night at home..

Your urine and blood tests today show that your kidneys are starting to get sick. There is a chance that they could get sicker in the future.

To help you with this goal, we will send a letter to your main doctor or community nurse outlining your risk factors, along with detailed suggestions about treatment if needed. In order to make sure your kidneys don't get sicker we need to make sure you are checked every year. We will recommend in that letter that your main doctor or nurse test your kidney function and blood pressure every year. To help them keep track of your kidney health, we will keep a record of your yearly kidney tests, and remind you and your main nurse or doctor if a test was missed.

We will also give you information about eating good and keeping active to help keep you and your kidneys healthy. We encourage you to follow-up and talk about eating good and being active with your doctor, community nurse, CHR and/or ADI worker.

(For WRTC area) Do you see a traditional healer? (If yes) you may want to talk with them about things you can do for kidney health or your overall health. If they give you medicine to use you may want to let the kidney doctor know that you are using traditional medicine.

Adult Scripts for Risk Based Counseling

NO RISK

No Current Risk:

The urine and blood tests done today show that your kidneys are healthy right now. Eating good food, cooking, being active, and not smoking will keep you and your kidneys healthy. We encourage you to learn more about staying healthy and to keep all your good habits. We will give you some information that may be helpful, and we encourage you to with your nurse, doctor, CHR and or ADI worker they have a lot of knowledge and information about things you can do to keep healthy
